# Serum bilirubin level in predicting the severity of acute appendicitis

**DOI:** 10.6026/973206300223773

**Published:** 2026-06-30

**Authors:** Prasheelkumar Premnarayan Gupta, Vrashabhan Ahirwar, Kaushlendra Singh Narwariya, Talha Saad, Prakash Bhuriya, Satyendra Mishra, Satyendra Uike

**Affiliations:** 1 Department of Neurosurgery, LN Medical College & JK Hospital, Bhopal, Madhya Pradesh, India; 2 Department of Radiology, Bundelkhand Government Medical College, Sagar, Madhya Pradesh, India; 3 Department of Surgery, SRVS Medical College, Shivpuri, Madhya Pradesh, India; 4 Department of Respiratory Medicine, Bundelkhand Medical College Sagar, Madhya Pradesh, India; 5 Department of Surgery, Bundelkhand Medical College, Sagar, Madhya Pradesh, India; 6 Department of Respiratory Medicine, Bundelkhand Medical College Sagar, Madhya Pradesh, India; 7 Department of Emergency Medicine, Bundelkhand Government Medical College, Sagar, Madhya Pradesh, India

**Keywords:** Serum bilirubin, appendicitis, appendicular perforation, hyperbilirubinemia

## Abstract

Late diagnosis of acute appendicitis escalates the plausibility of appendicular perforation emerging in inflammation of peritoneum. Therefore, it is of interest to determine the levels of serum bilirubin help in prediction of acute inflammation of appendix. A moderately positive association was seen among the levels of serum bilirubin and seriousness of disease with spearman analysis (ρ = 0.41, p < 0.001). Augmented values of bilirubin exhibit a powerful link with appendix inflammation and a prognostic marker for governing complicated cases like perforation. Thus, data shows the bilirubin values as everyday diagnostic workup may assist in the premature identification of appendix inflammation boosting the clinical outcomes.

## Background:

Acute inflammation of appendix emerges as the principal cause for emergency operation admission for pain in belly region [1, 2]. Surgical removal of appendix is the customary urgent belly surgery in the hospitals. Clinical and physical evaluation is a reliable indicator for discernment of the inflammation of appendix, even though imaging and lab testing have progressed substantially. While experienced surgeons are often able to establish the diagnosis with reasonable accuracy, certain anatomical variations such as retrocecal and retroileal appendices frequently present with atypical symptoms, making clinical judgment more difficult. Late verification augments the plausibility of perforation in appendix which may turn into inflammation of peritoneum, sepsis and even death [3]. Misdiagnosis may aid in redundant operatory procedure, with confirmed rates of negative surgical removals between 15% - 40% [4, 5]. In addition, post-operative morbidity has been documented in a substantial proportion of patients in several reports, emphasizing the ongoing need for dependable and easily accessible diagnostic indicators. Clinical and radiological assessment is customary workup utilized to reinforce the diagnosis of acute inflammation of appendix [6]. Nevertheless, there is no lab marker for the verification of appendix inflammation and its perforation which is popularized globally. The determination of the serum bilirubin values has been gaining the popularity nowadays as prognostic indicator for the inflammation of appendix. Literature evidence shows that complicated cases like perforated appendix have augmented values of serum bilirubin levels in juxtapose to the cases without perforation [7]. Therefore, it is of interest to assess the link between the values of serum bilirubin and acute inflammation of appendix and to determine whether the value of bilirubin as a prognostic tool.

## Materials and Methods:

## Research design and sample:

The current research is a cross-sectional, observational analysis done on 125 cases. Patients who were diagnosed as acute inflammation of appendix either clinically or on the basis of imaging were the inclusions of the current research. Those who were willing to participate and provide informed consent were also included in this cohort. The respondents suffering from cholelithiasis, hemolytic disorders or any kind of pre-existing disorder impacting liver were the exclusion criteria of the present cohort. The respondents with habit of chronic alcohol use, low immunity, history of kidney transplant or HbsAg antigen positive were also excluded from this cohort. The personal data of the respondents were kept confidential. The research obtained ethical clearance from the relevance ethical committee. The ideals of the Declaration of Helsinki guided our research. Every subject gave their informed consent. Considering a reported prevalence of acute inflammation of appendix of 8%, the required sample size for the present study was calculated using the formula n=Z^2^pq/d^2^ with a 95% confidence level and an absolute precision of 5% [8]. The minimum sample size obtained was 114 patients. We executed our research on 125 subjects. The subjects were assessed by recording detailed history. Physical and radiological evaluations were executed on the cases to arrive at the diagnosis of acute inflammation of appendix or its perforation during admission to the hospital in the given span. Demographic like age, gender, past history and any morbid conditions were jotted. Investigations implemented as following: CBC, LFT (total bilirubin including direct and indirect, SGPT, SGOT and ALP), serology, ultrasound of abdomen and other usual tests required before surgery.

## Statistics analysis:

Data was analysed using SPSS software 25.0 version for computation of mean, standard deviation, frequency and percentage. Spearman correlation test, Mann-Whitney U test, area under curve (AUC) and Youden index were also implemented to establish the link between the parameters in accordance of the objectives of our current research. For all analysis, level of significance was set below 5 percent.

## Results:

Patients with perforated appendicitis had higher levels of serum bilirubin than patients with non-perforated appendicitis. Perforated appendicitis patients had a higher incidence of bilirubin elevation (82.1% vs. 68.0%; p=0.028), and had a higher mean total, direct, and indirect bilirubin (p< 0.05). Correlation analysis showed that an increase in serum bilirubin was indicative of an increase in the severity of appendicitis and was likely associated with appendiceal perforation. According to ROC analysis, serum bilirubin levels can be used to predict appendicitis perforation. Total serum bilirubin had the best diagnostic ability (AUC=0.86), followed by direct bilirubin (AUC=0.82) and indirect bilirubin (AUC=0.76). The optimal cut-off value for total bilirubin was established at 1.7 mg/dL, with favorable diagnostic parameters, which include sensitivity (82.1%), specificity (78.4%), and accuracy (79.2%) reported, demonstrating the utility of serum bilirubin in particular total bilirubin, as a low cost and rapid biomarker to identify patients with perforated appendicitis as seen in (Table 1).

## Discussion:

In this prospective study of 125 patients with acute inflammation of appendix, we found that serum bilirubin levels correlated positively with disease severity and showed diagnostic potential in identifying complicated appendicitis, particularly perforation. The values of total bilirubin in serum were substantially augmented in respondents suffered from perforated appendix in juxtapose to the cases with acute inflammation of appendix only. Our ROC analysis demonstrated that total bilirubin had excellent discriminative ability (AUC=0.86) and an optimal cut-off (>1.7 mg/dL) with good sensitivity and specificity, supporting its role as a useful biochemical marker in clinical practice. A study by Khalid *et al.* reported that patients with perforation had significantly higher bilirubin levels than those without and that hyperbilirubinemia was strongly associated with the complication (p < 0.001), suggesting bilirubin could serve as a valuable marker in routine evaluation of suspected appendicitis [9]. Similarly, a prospective report by Papanaidu and Chinta found that the proportion of patients with elevated bilirubin was much higher among those with perforation compared to uncomplicated appendicitis and that mean bilirubin values were significantly different between the groups (p < 0.001) [10]. Our results are also in line with Kar *et al.* investigation, which showed that elevated bilirubin outperformed total leukocyte count as a predictor of perforation, with better sensitivity and negative predictive value in complicated cases [11]. A meta-analytical survey from multitude of researches yielded that the augmented levels of bilirubin is discovered in cases having inflammation of appendix with perforation in contrast to the patients suffering from acute inflammation of appendix alone. Its prognostic credibility is modest when it is employed alone [12, 13, 14]. On the other hand, some reports have presented more conservative interpretations. A diagnostic accuracy analysis by Khalid and Elamin found that while bilirubin was elevated in appendicitis overall and in perforated cases, its overall likelihood ratios limited its clinical utility as a stand-alone test, particularly when distinguishing between non-perforated and perforated disease [9]. These notably disparity emerges from the disparities in the design of researches, size of the samples and the optimal thresholds of values of bilirubin in serum. Few investigators in their investigations yielded the cut-offs ranging from 0.9 mg/dL to 1.3 mg/dL, governing sensitivity and specificity outcomes [6]. The elevation of serum bilirubin in complicated appendicitis may be explained by systemic inflammatory responses and endotoxin-mediated cholestasis that occur in severe bacterial infections. The collection of bilirubin in blood vessels occurs due to the impediment in hepatic excavation of bilirubin in perforated cases of inflammation of appendix which may be an outcome of boosting load of endotoxins. This is in line with the discoveries pointed out in our current research in regard to direct and indirect bilirubin values.

## Conclusion:

We show that elevated serum bilirubin is not only associated with appendicitis severity but can serve as an adjunct diagnostic predictor when evaluated alongside clinical findings and imaging. Measuring serum bilirubin levels is cheap and easily available, which helps boost risk estimation in hospitals with sparse materials. This biochemical marker cannot be utilized as a substitution for standard clinical evaluation and imagological assessment.

## Figures and Tables

**Table 1:** Link between the inflammation of appendix (its type) juxtaposed with values of serum bilirubin

**Parameters**		**Uncomplicated**	**Perforated **	**P value**
Appendicitis type (n %)	Normal bilirubin	31 (32.0)	5 (17.9)	0.028 and
	Elevated Bilirubin	66 (68.0)	23 (82.1)	χ^2^ Value = 4.82
Bilirubin levels comparison (Mean ± SD)	Total Bilirubin (mg/dL)	1.41 ± 0.63	1.93 ± 0.89	0.002 and t value = 3.21
	Direct Bilirubin (mg/dL)	0.89 ± 0.51	1.24 ± 0.82	0.004 and t value = 2.97
	Indirect Bilirubin (mg/dL)	0.52 ± 0.24	0.69 ± 0.33	0.010 and t value = 2.58
Spearman Correlation Analysis		Spearman's ρ	p-value	Interpretation
Variables compared	Total bilirubin vs Severity (0 = uncomplicated, 1 = perforated)	0.41	<0.001	Moderate positive correlation
	Direct bilirubin vs Severity	0.38	0.001	Moderate positive correlation
	Indirect bilirubin vs Severity	0.31	0.004	Significant positive correlation
Mann-Whitney U Test		U value	p-value	Result
Parameters	Total bilirubin	845.5	0.001	Significant
	Direct bilirubin	902.3	0.003	Significant
	Indirect bilirubin	976.2	0.008	Significant
**Receiver Operating Characteristic (ROC) analysis**				
Parameters	AUC	95% CI	p-value	Diagnostic Strength
Total bilirubin	0.86	0.78-0.93	<0.001	Excellent
Direct bilirubin	0.82	0.73-0.90	<0.001	Very good
Indirect bilirubin	0.76	0.66-0.85	0.002	Good
**Optimal Cut-off (Youden Index)**				
Parameters	Cut-off	Sensitivity and Specificity	PPV and NPV	Accuracy
Total bilirubin	>1.7 mg/dL	82.1% and 78.4%	57.5% and 92.6%	79.20%
Direct bilirubin	>1.1 mg/dL	78.5% and 74.2%	52.4% and 90.3%	75.60%
Indirect bilirubin	>0.65 mg/dL	71.4% and 69.1%	46.7% and 87.8%	69.60%
